# Theoretical and Experimental Research of Hydrogen Solid Solution in Mg and Mg-Al System

**DOI:** 10.3390/ma15051667

**Published:** 2022-02-23

**Authors:** Jinzhe Lyu, Roman R. Elman, Leonid A. Svyatkin, Viktor N. Kudiiarov

**Affiliations:** Division for Experimental Physics, School of Nuclear Science & Engineering, National Research Tomsk Polytechnic University, Lenin Ave. 43, 634050 Tomsk, Russia; czinchzhe1@tpu.ru (J.L.); svyatkin@tpu.ru (L.A.S.); kudiyarov@tpu.ru (V.N.K.)

**Keywords:** magnesium, aluminum, thin film, hydrogen, first-principle calculations

## Abstract

The study of hydrogen storage properties of Mg-based thin films is of interest due to their unique composition, interface, crystallinity, and high potential for use in hydrogen-storage systems. Alloying Mg with Al leads to the destabilization of the magnesium hydride reducing the heat of reaction, increases the nucleation rate, and decreases the dehydriding temperature. The purpose of our study is to reveal the role of the aluminum atom addition in hydrogen adsorption and accumulation in the Mg-H solid solution. *Ab initio* calculations of aluminum and hydrogen binding energies in magnesium were carried out in the framework of density functional theory. Hydrogen distribution and accumulation in Mg and Mg-10%Al thin films were experimentally studied by the method of glow-discharge optical emission spectroscopy and using a hydrogen analyzer, respectively. It was found that a hydrogen distribution gradient is observed in the Mg-10%Al coating, with more hydrogen on the surface and less in the bulk. Moreover, the hydrogen concentration in the Mg-10%Al is lower compared to Mg. This can be explained by the lower hydrogen binding energy in the magnesium-aluminum system compared with pure magnesium.

## 1. Introduction

As a high-energy-density and clean energy source, hydrogen energy has attracted increasing attention. The development and utilization of hydrogen energy involve the preparation, storage, transportation, and application of hydrogen. The storage of hydrogen energy is the key to expand the usage of hydrogen energy [1,2,3,4,5,6]. Among the numerous hydrogen storage materials [7,8,9,10], Mg is one of the most promising candidates due to its high hydrogen storage capacity up to 7.6 wt%, light weight, and low cost [11]. However, the main obstacle preventing the commercial use of magnesium as a hydrogen storage material is the low diffusion rate of hydrogen atoms in MgH_2_ layers [12,13]. Additionally, the activation energy of MgH_2_ formation can be overcome only at a high temperature up to 300 °C [11]. Tremendous efforts have been devoted to decreasing the diffusion barrier and hydrogenation temperature rate, including doping catalysts [14,15,16,17,18,19,20] and synthesis of nanostructured composites [20,21,22,23,24]. Such methods as melting, sintering, or mechanochemical synthesis by ball milling are used to achieve these goals for magnesium. Compared with the Mg-based hydrogen storage materials prepared by the methods mentioned above, Mg-based thin films show numerous advantages due to their interface, composition, and crystallinity being able to be accurately tailored on the nanoscale [25]. Thus, at present, many research groups continue their work on the study of the hydrogen storage properties of Mg-based thin films, for which one can refer to our previous review [26].

Doping Mg with Al leads to the formation of intermetallic compounds with a lower hydrogenation enthalpy in comparison with pure magnesium. It allows to destabilize magnesium hydride. Thus, alloying Mg with Al helps reduce the heat of reaction, increases the nucleation rate, and decreases the dehydrogenation temperature [27]. It should be noted that the Mg_17_Al_12_ phase formed during the hydrogenation process as a result of reaction between Al and Mg. The hydrogen absorption and desorption processes of the Mg_17_Al_12_ phase are completely reversible through multiple-step reactions [27]. The phase transformation of the γ–Mg_17_Al_12_ intermetallic compound during hydrogenation and dehydrogenation processes is reported by Andreasen [28]:

Mg_17_Al_12_ + 9H_2_ = 9MgH_2_ + 4Mg_2_Al_3_ ∆*H* = −74 kJ mol^−1^ H_2_ (1)

Mg_2_Al_3_ + 2H_2_ = 2MgH_2_ + 3Al ∆*H* = −71 kJ mol^−1^ H_2_ (2)

It is worth mentioning that Mg_2_Al_3_ would subsequently be transformed into MgH_2_ and Al only if higher hydrogenation pressure than that used for hydrogenation of Mg_17_Al_12_ were applied [29]. Although compared with transition metals, light metals cannot cause serious capacity loss [25], the solubility of H in solid Al is extremely low with atomic H/Al fractions in the range of 10^−6^ to 10^−8^ [30]. The density functional theory (DFT) calculation in the generalized gradient approximation method performed by Ismer et al. [31] showed that the formation energy for interstitial hydrogen is significantly lower in hcp Mg than in fcc Al, implying that the interstitial H concentration in Mg is more than nine orders of magnitude higher than in Al at room temperature.

In this paper, we theoretically studied hydrogen and aluminum binding energies in the Mg-Al-H system. The hydrogen accumulation in the magnesium and magnesium-aluminum system was experimentally investigated. The main aim of this work was to study the influence of the hydrogen and aluminum concentrations on their binding energies in the Mg-Al-H solid solution and to reveal the role of aluminum atom addition on hydrogen adsorption and accumulation in the Mg-H solid solution. The data obtained will be useful for further research of hydrogen storage materials [32,33,34].

## 2. Materials and Methods

### 2.1. Sample Preparation

Samples of stainless steel 12X18H10T with 20 mm × 20 mm × 1 mm dimensions were used as substrates. The deposition of coatings was carried out by the method of magnetron sputtering (physical vapor deposition). Sample preparation was carried out as follows: (1) grinding and polishing using silicon carbide abrasive paper (ISO from 160 to 4000); (2) exposure in the ultrasonic bath with acetone for 20 min; (3) additional cleaning of the surface with acetone before placing it in the chamber of the “Raduga-Spectrum” installation for ion-plasma spraying; (4) removing atomic layers from the surface by low-energy (2 keV) Ar^+^ ion bombardment for 20 min in the vacuum chamber of the “Raduga Spectrum” installation. After these procedures, the prepared steel substrates were coated on one side using a magnetron system.

Pure Mg and Mg-10%Al coatings were formed in a stationary mode using a magnetron sputtering process with a direct current source. Magnesium MG90 and Mg-10%Al custom-made targets (JSC NIIEFA, St. Petersburg, Russia) were used as cathodes for the magnetron system. Magnesium coating was deposited in argon atmosphere (0.12 Pa) at discharge voltage U = 450 V under a fixed sputter voltage of −600 V (1 min). Discharge current was I = 2.15 A and the deposition time was t = 15 min. Sputtering magnesium required a power of 1 kW. For Mg-10%Al coating discharge voltage was about 520 V, sputter voltage was −600 V (1 min), discharge current I = 2.25 A, and deposition time t = 17.5 min. Power (power discharge stabilization) for Mg-10%Al sputtering was 1.5 kW. All these parameters allowed to obtain a stable discharge and to ensure the required thickness of each type of coating.

### 2.2. Experimental Methods

S-3400N scanning electron microscope (Hitachi, Tokyo, Japan) was used to analyze the microstructure and thickness of the obtained coatings. The detailed elemental analysis was carried out using color mapping for the distribution of elements on the coatings surface. Structural phase analysis was performed on an XRD-7000S diffractometer equipped with a OneSight high-speed wide-angle detector (Shimadzu, Kyoto, Japan). Analysis of diffraction patterns and identification of phases were carried out using the PDF-4+2020 database and the PowderCell 2.4 program. The study of the distribution of elements in the coatings was carried out by the method glow-discharge optical emission spectroscopy (GDOES) on a GD-Profiler 2 spectrometer equipped with a high-frequency ac-powered pulse generator (Horiba, Kyoto, Japan).

An automated complex Gas Reaction Controller (Advanced Materials Research, Pittsburgh, PA, USA) was used to perform gas-phase hydrogenation of the coatings. Hydrogen pressure of about 30 atmospheres was used for hydrogenation. The heating rate was 6 °C/min and the maximum temperature was 400 °C. The samples were kept in a hydrogen atmosphere for 12 h. The hydrogen analyzer RHEN602 (LECO, St. Joseph, CA, USA) was used to determine the hydrogen content in the coatings. The studies performed using all the above-mentioned equipment were conducted on the premises of Tomsk Polytechnic University.

### 2.3. Ab Initio Calculations

**Figure 1:**
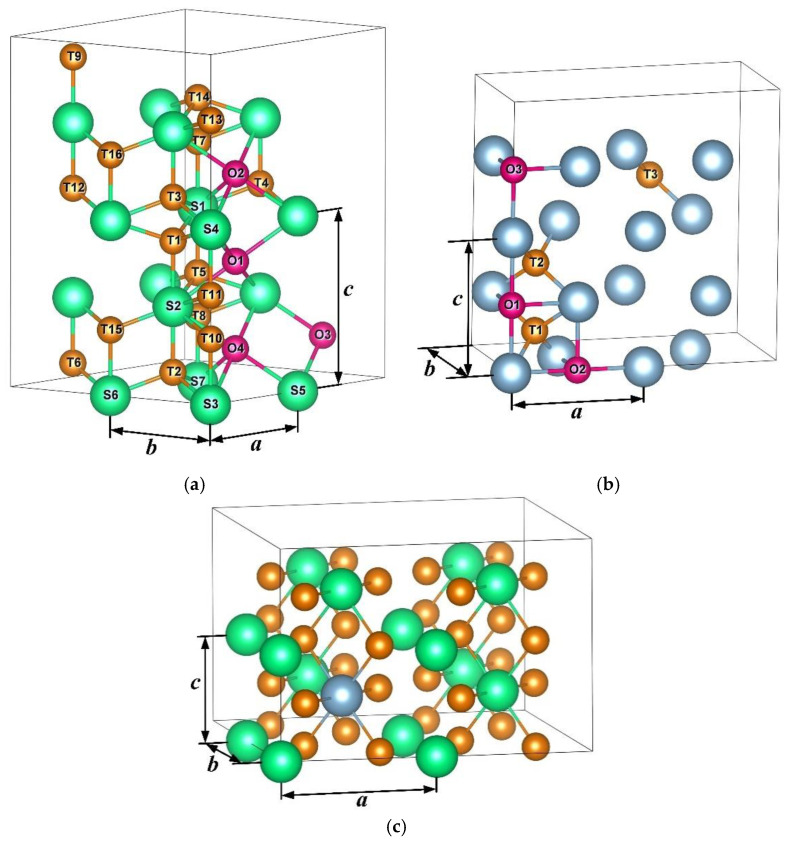
(**a**) Positions of the considered substitution sites for Al atoms and positions of the considered interstitial sites for H atoms in the hcp Mg_16_ supercell; (**b**) positions of the considered interstitial sites for H atoms in the fcc Al_16_ supercell; (**c**) positions of the considered substitution sites for Al atoms in the bct Mg_16_H_32_ supercell. Magnesium atoms are green, aluminum atoms are blue, tetrahedral sites are orange, and octahedral sites are pink.

Self-consistent calculations of the total energy of a pure Mg and Al, a molecule H_2_ and Mg-H, Al-H, and Mg-Al-H solid solutions were carried out within the density functional theory using the optimized norm-conserving Vanderbilt pseudopotential method [35], as implemented in the ABINIT code [36,37]. The exchange and correlation effects were described within the generalized gradient approximation in the form of Perdew–Burke–Ernzerhof (PBE) [38]. The cutoff energy for the plane-wave basis was set to 816 eV. The k-point mesh in the structural optimization were set to 14 × 14 × 9 for hcp Mg supercell, 12 × 12 × 12 for fcc Al supercell, 5 × 5 × 3 for hcp Mg_16−*x*_Al*_x_*H*_y_* (*x* = 0, 1, 2, 3 and *y* = 0, 1, 2) supercell (Figure 1a), 6 × 12 × 6 for fcc Al_16_H*_x_* (*x* = 0, 1, 2) supercell (Figure 1b) and 5 × 5 × 8 for bct Mg_16−*x*_Al*_x_*H_32_ (*x* = 0, 1) supercell (Figure 1c). The atoms in the system were assumed to be in the equilibrium configuration when the force on each atom was below 5 meV/Å. The hcp Mg_16−*x*_Al*_x_*H*_y_* solid solution model was built with Al in the substitution sites and hydrogen in tetrahedral (T) or octahedral (O) interstitial sites of the supercell consisting of 2 × 2 × 2 hcp Mg unit cell. The fcc Al_16_H*_x_* solid solution model was built with hydrogen in tetrahedral (T) or octahedral (O) interstitial sites of the supercell consisting of 2 × 1 × 2 fcc Al unit cell. The bct Mg_16−*x*_Al*_x_*H_32_ model was built with Al in the substitution sites of Mg_16_H_32_ supercell consisting of 2 × 2 × 2 bct Mg_2_H_4_ unit cell. For a more convenient discussion of results, the T and O sites in Figure 1 are enumerated.

To analyze the structural stability of the systems under consideration, the binding energies of aluminum ($E_{\mathrm{Al}}^{b}$) and hydrogen ($E_{H}^{b}$) in the Mg-Al-H system were calculated:

(3) $$E_{\mathrm{Al}}^{b}=\frac{E_{\mathrm{tot}}\left({\mathrm{Mg}}_{16}H_{y}\right)+\frac{x}{16}E_{\mathrm{tot}}\left({\mathrm{Al}}_{16}\right)-E_{\mathrm{tot}}\left({\mathrm{Mg}}_{16-x}{\mathrm{Al}}_{x}H_{y}\right)-\frac{x}{16}E_{\mathrm{tot}}\left({\mathrm{Mg}}_{16}\right)\;}{x},$$

(4) $$E_{H}^{b}=\frac{E_{\mathrm{tot}}\left({\mathrm{Mg}}_{16-x}{\mathrm{Al}}_{x}\right)+\frac{y}{2}E_{\mathrm{tot}}\left(H_{2}\right)-E_{\mathrm{tot}}\left({\mathrm{Mg}}_{16-x}{\mathrm{Al}}_{x}H_{y}\right)}{y},\;$$

Here, $E_{\mathrm{tot}}\left({\mathrm{Al}}_{16}\right)$ and $E_{\mathrm{tot}}\left({\mathrm{Mg}}_{16}\right)$ are the total energies of pure aluminum and magnesium in the presence of 16 aluminum atoms in the fcc supercell or 16 magnesium atoms in the hcp supercell, respectively;

$E_{\mathrm{tot}}\left(H_{2}\right)$ is the total energy of the hydrogen molecule;

$E_{\mathrm{tot}}\left({\mathrm{Al}}_{16}H_{y}\right)$ and $E_{\mathrm{tot}}\left({\mathrm{Mg}}_{16}H_{y}\right)$ are the total energies of the fcc Al-H and the hcp Mg-H solid solutions supercell, respectively;

$E_{\mathrm{tot}}\left({\mathrm{Mg}}_{16-x}{\mathrm{Al}}_{x}\right)$ and $E_{\mathrm{tot}}\left({\mathrm{Mg}}_{16-x}{\mathrm{Al}}_{x}H_{y}\right)$ are the total energies of the hcp Mg-Al solid solution supercell and the Mg-Al-H supercell;

*x* and *y* are the numbers of Al and H atoms, respectively, in the Al-H, Mg-H, Mg-Al, and Mg-Al-H supercells (*x* = 0, 1, 2, 3, 16, *y* = 0, 1, 2, 32).

To analyze the influence of the H and Al atoms on the lattice constants of the hcp Mg matrix, the average lattice constants were calculated:

(5) $$\bar{a}=\frac{\sum_{N}a+\sum_{N}b}{2N}$$

(6) $$\bar{c}=\frac{\sum_{N}c}{N}$$

Here, *a*, *b*, *c* are the calculated lattice constants of hcp Mg matrix shown in Figure 1a for a certain calculation configuration;

*N*—the number of calculation configurations for a certain system.

## 3. Results and Discussion

### 3.1. First-Principles Calculations of Mg-Al-H System

First of all, geometry optimization of the H_2_ molecule and the hcp Mg and fcc Al bulk structures was conducted. The value of the total energy of the H_2_ molecule was calculated to be −31.729 eV, very close to the value of −31.565 eV obtained by using the von Barth–Hedin exchange-correlation potential [39]. The lattice constants calculated for pure Mg and Al (Table 1) are in good agreement with the results of experimental research [40,41] and other theoretical studies [41,42,43]. Thus, the chosen computation parameters and the model can provide a reliable description of the Al-H, Mg-H, Mg-Al, and Mg-Al-H solid solutions.

Analyzing the results presented in Table 2, we can deduce that compared with Al-H solid solution, the Mg-H solid solution forms easier since the binding energy of Mg_16_H and Mg_16_H_2_ is larger than that of Al_16_H and Al_16_H_2_, respectively. The difference in the H binding energy in the Al-H and Mg-H solid solutions can be explained by three factors [31]: first, the influence of the lattice type (fcc in Al versus hcp in Mg); second, the larger equilibrium volume in Mg compared to Al; and third, the difference in the valence electron number (Mg has one less than Al). It is reported in [11] that the continuous Al layer on Mg in the Mg/Al film system prevents hydrogen diffusion towards the Al-Mg interface at room temperature, as a result, the MgH_2_ phase is not formed. We believe that the blocking effect of the continuous Al layer does not arise from the diffusion of hydrogen inside the Al layer. Some justification for this hypothesis can be found in the fact that hydrogen diffusivity in Al (at 300 K) is similar to Mg or slightly improved [28]. Considering the larger binding energy of Mg_16_H and Mg_16_H_2_ than that of Al_16_H and Al_16_H_2_, it can be believed that the blocking effect of the continuous Al layer is mainly caused by the difficulty in the formation of the Al-H solid solution since for the Mg/Al film system the diffusion of hydrogen atoms through the continuous Al layer on Mg towards Al-Mg interface occurs only when hydrogen is solid-dissolved in Al. It was reported that a 1 nm Al interlayer grows discontinuously on magnesium, forming isolated Al islands which are less likely to form an alloy with Mg and therefore serve as heterogeneous nucleation centers to collect hydrogen atoms [11,25]. According to the above discussion, the higher hydrogen storage capacity of the Mg-Al alloy film system can be attributed to the following factors: (1) isolated Al islands can be formed on the surface of Mg particle. It can be proposed that the volume expansion from Al to Al-H solid solution, as shown in Table 1 and Table 2, leads to the volume expansion of the attached Mg lattice, which allows dispelling the accumulated elastic strain caused by the around 20% lattice expansion from the initial Mg metal to the rutile-type tetragonal phase of MgH_2_ [12], making the nucleation and growth of MgH_2_ fast and easy; (2) the blocking effect of the MgH_2_ layer can be weakened as a result of the easier hydrogen diffusion through Al islands or through the additional grain boundaries between MgH_2_ and Al islands than through MgH_2_; (3) the dispersed Mg_17_Al_12_ alters the hydrogenation pathway, which decreases the heat of formation of MgH_2_. It is also believed that Mg_17_Al_12_ acts as a catalyst to decrease the dissociation energy of H_2_ and improve the hydrogen sorption kinetics of Mg [44,45]. In Mg/Al film system, thermodynamically, compared with Mg-H solid solution, the formation of Al-H solid solution is more unfavorable, thus it is difficult for hydrogen to enter the lattice of the continuous Al layer on Mg and thereby diffuse on the Al-Mg interface or the surface of the Mg_17_Al_12_ phase, leading to a hydrogen storage capacity even lower than pure Mg film.

The formation of AlH_3_ was not observed during the hydrogenation of Mg-Al in our experiment. There are two explanations for this: (1) the AlH_3_ formation is possible at high hydrogen pressures (more than 25 kbar) [28,46,47]; (2) the temperatures used to achieve acceptable kinetics for MgH_2_ exceed the decomposition temperature of AlH_3_ since AlH_3_ can be decomposed into Al and H_2_ at 170 °C, and the decomposition enthalpy is only 10 kJ mol^−1^ H_2_ [48].

**Figure 2:**
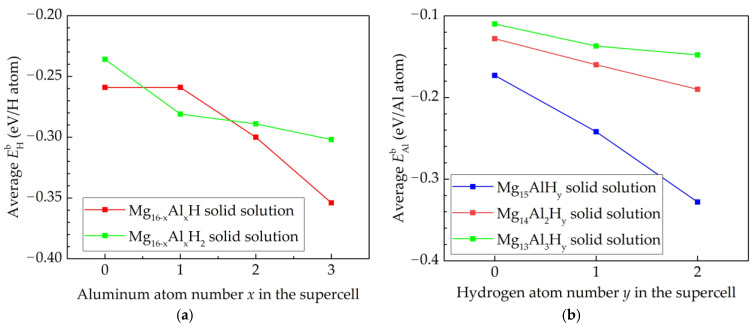
(**a**) Effect of Al atom on the average $E_{H}^{b}$ of hcp Mg_16−*x*_Al*_x_*H*_y_* solid solution (*x* = 0, 1, 2, 3, *y* = 1, 2); (**b**) effect of H atom on the average $E_{\mathrm{Al}}^{b}$ of hcp Mg_16−*x*_Al*_x_*H*_y_* solid solution (*x* = 1, 2, 3, *y* = 0, 1, 2).

From Table 3 and Figure 2, it can be seen that the increase of H atoms in the Mg lattice will reduce $E_{\mathrm{Al}}^{b}$. This is due to the fact that in the Mg-H solid solution, Mg-H bonds are formed, which are stronger than Mg-Mg bonds, leading to the more difficult substitution of Mg atoms by Al atoms. The reduced $E_{H}^{b}$ with the increase of Al atoms in the Mg lattice can be explained by the weaker Al-H bonds than Mg-H bonds. The same conclusion can also be used for Al-doped bct Mg hydride because the calculated results $E_{\mathrm{Al}}^{b}$(Mg_15_Al) = −0.173 eV/Al atom, $E_{\mathrm{Al}}^{b}$(Mg_15_AlH_32_) = −1.779 eV/Al atom, $E_{H}^{b}$(Mg_16_H_32_) = 0.268 eV/H atom, and $E_{H}^{b}$(Mg_15_AlH_32_) = 0.218 eV/H atom show the reduction of $E_{\mathrm{Al}}^{b}$ with the increase of H atoms in the Mg lattice and the reduction of $E_{H}^{b}$ with the increase of Al atoms in the Mg lattice.

It was reported that the relationship between lattice constants (Å) and solubility of Mg-Al solid solution satisfied the following empirical formula [49]:

(7) $$a=2.807+4.0234\times 10^{-3}\times \left(100-z\right)$$

(8) $$c=4.672+5.3864\times 10^{-3}\times \left(100-z\right)$$

where *z* represents the mole solubility of Al, at.%. For Mg_15_Al, Mg_14_Al_2_, and Mg_13_Al_3_ the mole solubility of Al are 6.25 at.%, 12.5 at.%, and 18.75 at.% respectively, the corresponding lattice constants was calculated to be *a*(Mg_15_Al) = 3.184 Å, *c*(Mg_15_Al) = 5.177 Å, *a*(Mg_14_Al_2_) = 3.159 Å, *c*(Mg_14_Al_2_) = 5.143 Å, *a*(Mg_13_Al_3_) = 3.134 Å, *c*(Mg_13_Al_3_) = 5.110 Å by this empirical method, close to the average value of lattice constants calculated by Equations (5) and (6) ($\bar{a}$(Mg_15_Al) = 3.201 Å, $\bar{c}$(Mg_15_Al) = 5.143 Å, $\bar{a}$(Mg_14_Al_2_) = 3.155 Å, $\bar{c}$(Mg_14_Al_2_) = 5.065 Å, $\bar{a}$(Mg_13_Al_3_) = 3.151 Å, $\bar{c}$(Mg_13_Al_3_) = 5.026 Å), which again validates the computation details.

**Figure 3:**
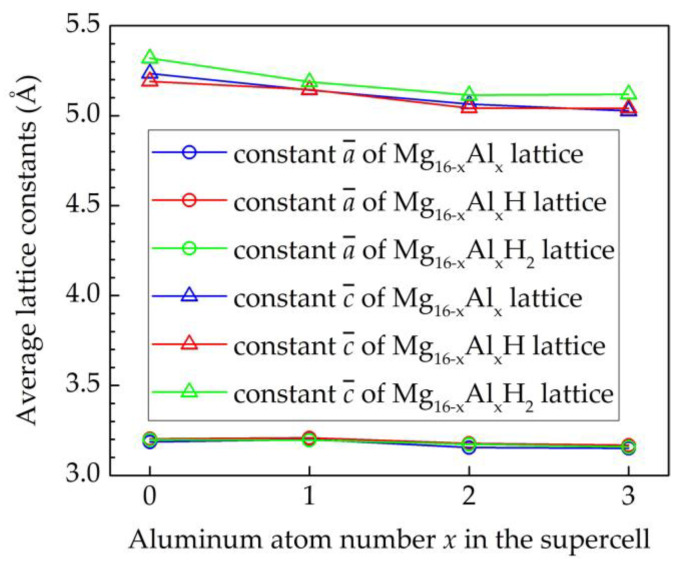
Dependence of the lattice constants of hcp Mg_16−*x*_Al*_x_*H*_y_* (*x* = 0, 1, 2, 3, *y* = 0, 1, 2) on Al and H atoms.

From Figure 3, it can be seen that increasing the number of aluminum atoms in the hcp Mg lattice slightly decreases the parameter *c*, while adding two H atom in the hcp Mg lattice slightly increases this parameter. The addition of Al and H atoms to the hcp Mg lattice has almost no effect on its constant *a*.

### 3.2. Experimental Research of Mg-Al-H System

**Figure 4:**
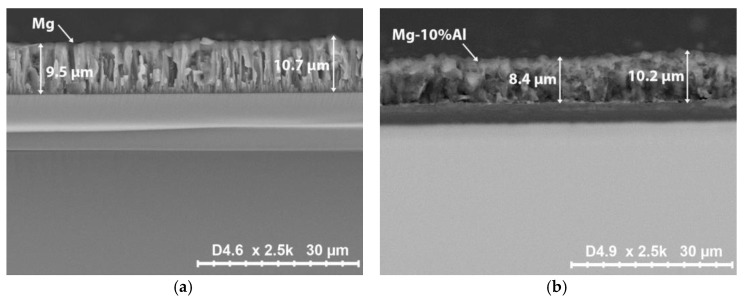
SEM images of a transverse cleavage of (**a**) Mg coating; (**b**) Mg-10%Al coating.

Images of a transverse cleavage are shown in Figure 4. Analysis of the images showed that the coatings of pure magnesium have a pronounced columnar structure with the presence of intergranular pores (Figure 4a).

**Figure 5:**
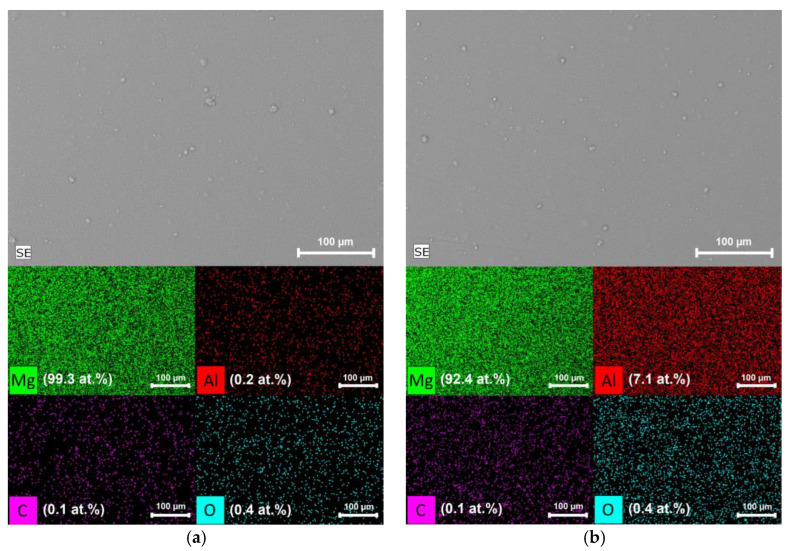
SEM images and elemental mapping from the surface of (**a**) Mg coating; (**b**) Mg-10%Al coating.

At the same time, magnesium coatings have a fairly uniform thickness with deviations within ±600 nm. The coatings obtained from the Mg-10%Al alloy have a more porous microstructure, which is caused by a more uneven grain growth and, as a consequence, a higher thickness heterogeneity (Figure 4b). Thickness deviations for Mg-10%Al coating were ±900 nm. The detailed elemental analysis was carried out using color mapping for the distribution of elements on the coatings surface. Figure 5a,b shows the representative scanning electron micrograph of Mg and Mg-10%Al coatings, respectively, with their corresponding color mapping.

In both cases, microparticles of the sprayed material are observed on the surface; however, the main area of the coatings is a plateau. It is clear from the color mapping of the pure magnesium coating that Mg is homogeneously distributed on the surface. O and C are present in small amounts and are concentrated in the unevenness of the coating relief. Al is not present on the coating surface. Element distribution maps for Mg-10%Al coating indicate a homogeneous distribution of Mg and Al on the surface. Elements such as O and C are also present in very small amounts.

**Figure 6:**
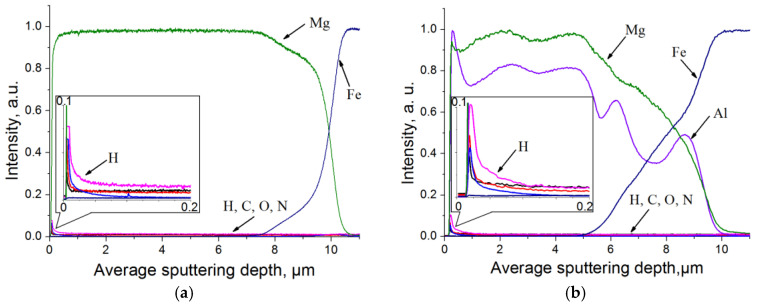
Depth distribution of chemical elements for samples with (**a**) Mg coating; (**b**) Mg-10%Al coating. The insets represent the hydrogen distribution in the coatings in more detail.

Figure 6 shows the results of elements distribution investigation in the samples with (a) Mg coating; (b) Mg-10%Al coating. Analyzing depth distributions of the different chemical elements shown in Figure 6, it can be concluded that the Mg and Mg-10%Al coatings were applied uniformly. It also has to be noted that, due to the formation of a multiphase system, irregularities in the luminescence intensities are observed for samples with Mg-10%Al coating. The total thickness of the Mg and Mg-10%Al coatings was about 10 µm.

For both samples, it can be seen that hydrogen is not observed either in the coating or in the metal substrate. A small amount of hydrogen is contained on the surface of the coatings (the insets in Figure 6). This may be due to surface contamination as well as the presence of these gases in the atmosphere.

To determine the hydrogen content in the coatings, the method of extraction in an inert gas medium was used, which was carried out using a LECO RHEN602 gas analyzer. The relative error of this method is ±2.5%. The hydrogen content of the sample coated with magnesium and Mg-10%Al was 7.4 ppm and 5.3 ppm, respectively. This indicates that there is no hydrogen present in the samples.

**Figure 7:**
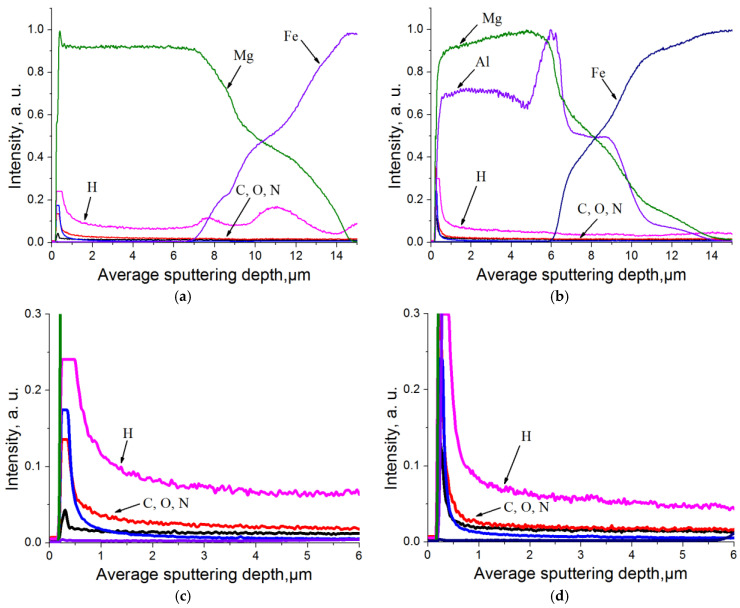
Depth distribution of chemical elements for samples with (**a**) Mg coating; (**b**) Mg-10%Al coating after hydrogenation during 10 h and 30 atm. The figures (**c**,**d**) represent the hydrogen distribution in the coatings in more detail.

The depth distributions of the different chemical elements for samples after hydrogenation is shown in Figure 7.

It can be seen that a certain amount of hydrogen is contained on the surface of the coatings. In addition, for a magnesium coating, hydrogen is uniformly distributed in the coating, and an increased concentration of hydrogen is observed at the coating–substrate interface (Figure 7c). This may be due to the formation of voids or other defects in which hydrogen accumulates. Thus, the uniform distribution of hydrogen in the coating indicates the penetration of hydrogen atoms into the bulk of the coating. For the Mg-10%Al sample, a hydrogen distribution gradient in the coating is observed (Figure 7d). This is consistent with theoretical calculations. Aluminum inhibits hydrogen diffusion due to the less favorable condition for hydrogen to be in the magnesium-aluminum system. The hydrogen content of the sample coated with magnesium and Mg-10%Al after hydrogenation was 13 ppm and 10 ppm, respectively.

## 4. Conclusions

Analysis of the obtained experimental data showed that a hydrogen distribution gradient is observed in the magnesium-aluminum coating, with more hydrogen on the surface and less in the bulk. In addition, the hydrogen content in the magnesium-aluminum system is lower compared to pure magnesium. This is due to the fact that the hydrogen binding energy in the magnesium-aluminum system is significantly lower compared to pure magnesium. This leads to the fact that it is less favorable for hydrogen to be in the magnesium-aluminum system; therefore, hydrogen accumulates on the surface during hydrogenation, while the diffusion of hydrogen into the bulk of the magnesium-aluminum system occurs more slowly compared to pure magnesium. In addition, it was revealed that increasing the aluminum and hydrogen concentrations in the Mg-Al-H solid solution slightly distort the hcp Mg lattice along the hexagonal axis and has almost no effect on the lattice constant in the basal plane. Thus, we can conclude that on the basis of theoretical and experimental studies, the accumulation of hydrogen in the form of a solid solution is more preferable in pure magnesium than in magnesium with aluminum. However, it is of interest to conduct such studies on the effect of aluminum on the hydrogen accumulation in magnesium hydrides.

## Figures and Tables

**Table 1:** Lattice constants of fcc Al and hcp Mg.

Elements	Lattice Constants, Å		
	This Work	Experiments	Other Calculations
Al	*a* = 4.04	*a* = 4.05 [40]	*a* = 4.021 [41] *a* = 3.982 [42]
Mg	*a* = 3.186, *c* = 5.235	*a* = 3.21, *c* = 5.213 [41]	*a* = 3.19, *c* = 5.17 [43] *a* = 3.192, *c* = 5.206 [41]

**Table 2:** The binding energy of Al_16_H*_y_* and Mg_16_H*_y_*. $E_{calc}^{f}$ is the calculated formation energy from previous theoretical studies for comparison.

System	Lattice Constants, Å			Site of H Atom	$\mathrm{Binding}\;\mathrm{Energy}\;E_{H}^{b},\;\mathrm{eV}/H\;\mathrm{Atom}$	Other Calculated Formation Energy, eV/H Atom
	*a*	*b*	*c*			
Mg_16_H	3.203	3.203	5.196	T	−0.190	${\mathrm{Mg}}_{48}H,\;E_{calc}^{f}$ = 0.12 ^a^
	3.204	3.204	5.186	O	−0.327	${\mathrm{Mg}}_{48}H,\;E_{calc}^{f}$ = 0.26 ^a^
Mg_16_H_2_	3.211	3.211	5.269	T1, T2	−0.204	-
	3.172	3.172	5.427	T1, T3	−0.299	-
	3.213	3.214	5.262	T2, T4	−0.204	-
Al_16_H	4.059	4.037	4.059	O	−0.789	${\mathrm{Al}}_{32}H,\;E_{calc}^{f}$ = 0.77 ^a^
	4.053	4.071	4.053	T	−0.680	${\mathrm{Al}}_{32}H,\;E_{calc}^{f}$ = 0.69 ^b^; ${\mathrm{Al}}_{32}H,\;E_{calc}^{f}$ = 0.68 ^a^
Al_16_H_2_	4.083	4.088	4.067	O1, O2	−0.748	-
	4.080	4.083	4.080	O1, O3	−0.789	-
	4.083	4.059	4.083	T1, T2	−0.626	-
	4.072	4.077	4.072	T1, T3	−0.721	-
	4.083	4.088	4.067	O1, T1	−0.707	-
	4.080	4.083	4.080	O1, T3	−0.721	-

^a^ Reference (DFT GGA) [31]; ^b^ Reference (DFT GGA) [30].

**Table 3:** The binding energy of hcp Mg_16−*x*_Al*_x_*H*_y_*, *x* = 1, 2, 3, *y* = 0, 1, 2.

System	Lattice Constants, Å			Substitution Site of Al Atom	Site of H Atom	Binding Energy	
	*a*	*b*	*c*			$E_{Al}^{b},\;\mathrm{eV}/\mathrm{Al}\;\mathrm{Atom}$	$E_{H}^{b},\;\mathrm{eV}/H\;\mathrm{Atom}$
Mg_15_Al	3.201	3.201	5.143	S1	-	−0.173	-
Mg_14_Al_2_	3.168	3.169	5.044	S1, S2	-	−0.133	-
	3.126	3.126	5.108	S1, S3	-	−0.146	-
	3.169	3.169	5.043	S1, S4	-	−0.105	-
Mg_13_Al_3_	3.172	3.172	5.004	S5, S6, S7	-	−0.083	-
	3.131	3.130	5.048	S1, S2, S6	-	−0.137	-
Mg_15_AlH	3.211	3.211	5.141	S1	T5	−0.282	−0.299
	3.206	3.206	5.149	S1	T6	−0.201	−0.218
Mg_15_AlH_2_	3.166	3.166	5.342	S1	T5, T7	−0.310	−0.272
	3.167	3.254	5.076	S1	O1, O2	−0.364	−0.299
	3.211	3.211	5.142	S1	T6, O3	−0.310	−0.272
Mg_14_Al_2_H	3.173	3.173	5.064	S1, S4	T5	−0.173	−0.327
	3.169	3.169	5.067	S1, S4	T8	−0.133	−0.245
	3.174	3.174	5.063	S1, S4	T2	−0.119	−0.218
	3.200	3.200	5.004	S1, S4	O3	−0.187	−0.354
	3.176	3.176	5.010	S1, S4	O4	−0.187	−0.354
Mg_14_Al_2_H_2_	3.168	3.204	5.125	S1, S4	T5, T1	−0.214	−0.313
	3.174	3.174	5.041	S1, S4	O3, T9	−0.133	−0.231
	3.168	3.168	5.147	S1, S4	T5, T7	−0.214	−0.313
	3.167	3.167	5.135	S1, S4	T10, T7	−0.160	−0.259
	3.172	3.172	5.122	S1, S4	T11, T8	−0.228	−0.327
Mg_13_Al_3_H	3.164	3.169	5.043	S5, S6, S7	T2	−0.164	−0.435
	3.169	3.169	5.039	S5, S6, S7	T12	−0.110	−0.272
Mg_13_Al_3_H_2_	3.167	3.167	5.072	S5, S6, S7	T10, T13	−0.083	−0.204
	3.136	3.136	5.144	S5, S6, S7	T8, T14	−0.183	−0.354
	3.160	3.204	5.067	S5, S6, S7	T8, T15	−0.192	−0.367
	3.171	3.172	5.118	S5, S6, S7	T7, T16	−0.146	−0.299
	3.133	3.133	5.194	S5, S6, S7	T5, T7	−0.137	−0.286

## Data Availability

The raw/processed data required to reproduce these findings cannot be shared at this time as the data also forms part of an ongoing study.
